# County-Level Integrated Healthcare Practice in China: A Kaiser Permanente-Inspired Approach

**DOI:** 10.5334/ijic.8610

**Published:** 2024-12-31

**Authors:** Na Li, Yin Dong, Gaofeng Zhang

**Affiliations:** 1 Ningbo Insight Hospital Management Consulting, No. 41 Xingning Road, Ningbo, Zhejiang Province, CN; 2 Yuhuan People’s Hospital, No. 18 Changle Road, Yuhuan, Taizhou City, Zhejiang Province, CN

**Keywords:** integrated care, county-level practice, population health management

## Abstract

**Introduction:**

China’s rapidly aging population and rise in chronic diseases put immense strain on the country’s healthcare system. To address these challenges, Yuhuan People’s Hospital established County-level Integrated Health Organization (CIHO) as part of the Healthy China 2030 initiative.

**Description:**

Based on the Kaiser Permanente (KP) model, the CIHO takes a multi-disciplinary, collaborative approach to deliver integrated care. It brings together various medical specialties, collaborates with community organizations and companies, and implements reforms in information technology and payment models. Through these efforts, the CIHO has significantly improved healthcare delivery in Yuhuan county.

**Discussion:**

Population segmentation relies on data integration and segmentation tools to identify targeted healthcare needs. The allocation and collaboration of health workforce for residents with different health conditions are suggested to be dynamically designed according to both internal and external factors. Corresponding payment mechanism is also an important factor that needs to be taken into consideration.

**Conclusion:**

The CIHO’s success has provided a model for integrated, efficient healthcare that could be replicated in other regions of China and offer insights for rural areas in other countries facing similar demographic and epidemiological pressures.

## Introduction

The escalating burden of healthcare needs, particularly for chronic conditions and multi-morbid patients, underscores the imperative for a more cohesive and efficient healthcare delivery system. Integrated care, which centers on people’s healthcare needs through the redesign of healthcare delivery systems across various settings by a coordinated multidisciplinary team, has emerged as a promising approach to address the mismatch between complex healthcare needs and fragmented health services [1,2,3]. Its implementation yields benefits, including optimized resource utilization, enhanced care continuity, and proactive health management. This approach not only responds to current demands but also strategically positions itself as a forward-looking strategy to build resilient healthcare systems [4,5].

Globally, countries have actively explored diverse approaches to integrated care, such as the chronic care model, individual care plans, and integrated care in the Basque Country [6,7,8]. In recent years, research teams have investigated integrated care models for specific diseases, including dementia, depression, and atrial fibrillation [9,10,11]. The World Health Organization (WHO) categorizes these practices into three levels: individual models, group and disease-specific models, and population-based models, providing experiential references for policymakers and health managers across diverse settings [1]. However, due to heterogeneity in healthcare system design, stages of economic development, and sociocultural factors among countries and regions, successful practices can only serve as guiding principles, as no single model can be a panacea for all [12,13].

In 2016, the report “Deepening Health Reform in China”, published by the WHO, World Bank, and the Chinese government, introduced the concept of people-centered integrated care for the first time [14]. In 2017, the General Office of the State Council issued guiding opinions on promoting the construction and development of medical consortia, suggesting four types: hospital groups in urban areas, medical associations in rural areas, cross-regional specialist alliances, and tele-collaboration networks in remote areas [15]. In 2018, Zhejiang Province launched the establishment of medical associations, mandating each county to establish 1–3 medical associations based on local situations, with the aim of integrating resources from county and township health institutions [16]. However, this top-down policy lacked a corresponding integrated payment mechanism and a tailored information system, resulting in limited improvements in providing coordinated care [17,18].

The County-level Integrated Health Organization (CIHO) of Yuhuan People’s Hospital (abbreviated as Yuhuan First CIHO), located in Zhejiang province, has gradually become a CIHO template in China. After five years of robust development, Yuhuan First CIHO was awarded the highest grade of ‘A++’ in the initial round of the 2022 national assessment for compact medical associations. In contrast to the Luohu model, which represents urban practices [19], Yuhuan First CIHO represents a county-level integrated care approach, incorporating innovative methods such as the application of new technologies and the involvement of social organizations. Therefore, this paper aims to: (1) introduce the practices of Yuhuan First CIHO; (2) evaluate its construction outcomes; and (3) summarize implementation lessons and key considerations, offering insights for rural areas that are exploring and promoting integrated healthcare services.

## Background

**Figure 1:**
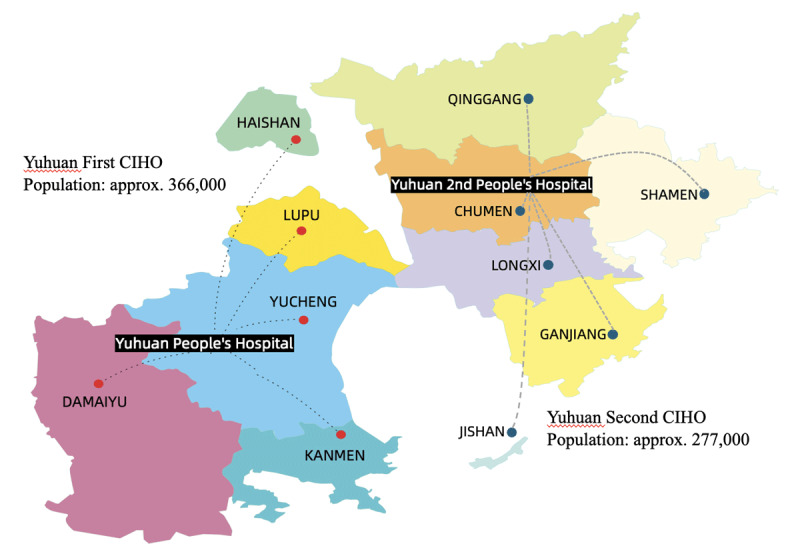
The map of YUHUAN.

Situated in the southeastern part of Zhejiang Province, Yuhuan is a typical coastal county covering a total area of 2,279 square kilometers, of which 378 square kilometers are land. The city features a peninsula and several islands, the main island of which is connected to the peninsula by a bridge (Figure 1). As of 2023, the permanent resident population reached 643,000, with a per capita Gross Domestic Product of approximately $16,349 which is equivalent to 129 percent of the national average. The numbers of hospital beds, practicing physicians and registered nurses per thousand residents are 3.66, 2.56 and 2.76 respectively. The allocation of medical resources is far lower than the average level of Zhejiang province (6.13, 4.01, 4.41 respectively) and the country (7.24, 3.39, 3.99 respectively), which puts higher requirements on the efficiency of health service delivery. Remarkably, the participation rate in basic medical insurance has reached an impressive 99.90%.

In 2018, Yuhuan established two CIHOs. Yuhuan First CIHO, encompassing the main island, includes Yuhuan People’s Hospital and five Township Health Centers (THCs). Meanwhile, the CIHO of Yuhuan Second People’s Hospital, covering the peninsula, comprises Yuhuan Second People’s Hospital and six THCs. Diverging from the scope of medical associations, the primary objective of CIHOs was to underscore the concept of health-centered care [17]. This involves integrating resources from various sectors to optimize health service processes and enhance residents’ well-being.

**Figure 2:**
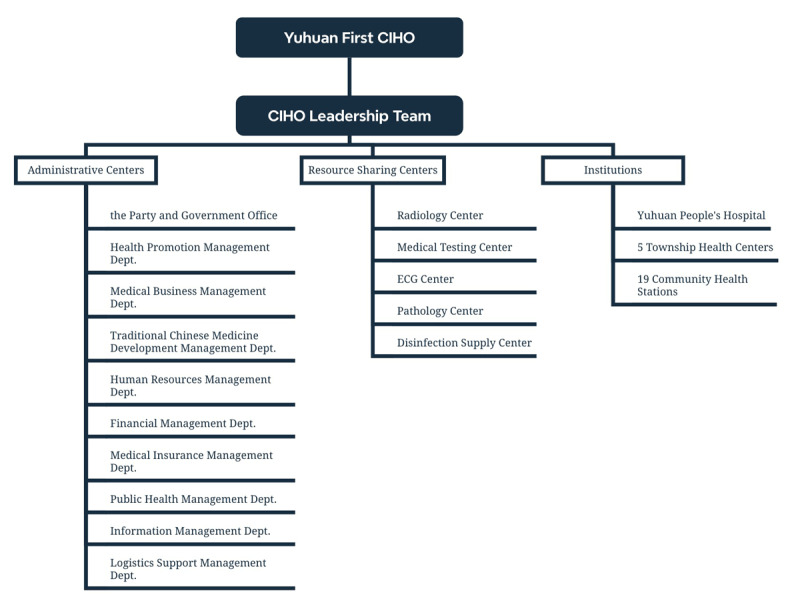
The organizational structure of Yuhuan First CIHO.

Yuhuan First CIHO caters to approximately 366,000 residents and boasts a capacity of 888 beds. In 2018, to enhance governance efficiency, a leadership team was established, overseeing ten administrative centers and five resource sharing centers (Figure 2). This organizational structure facilitates standardized administration and unified operations across 29 centers, encompassing administrative management, medical operations, logistical services, and information systems. In 2023, the CIHO delivered services for 2.79 million outpatient visits and recorded 40,961 discharges.

## The Kaiser Permanente model

**Figure 3:**
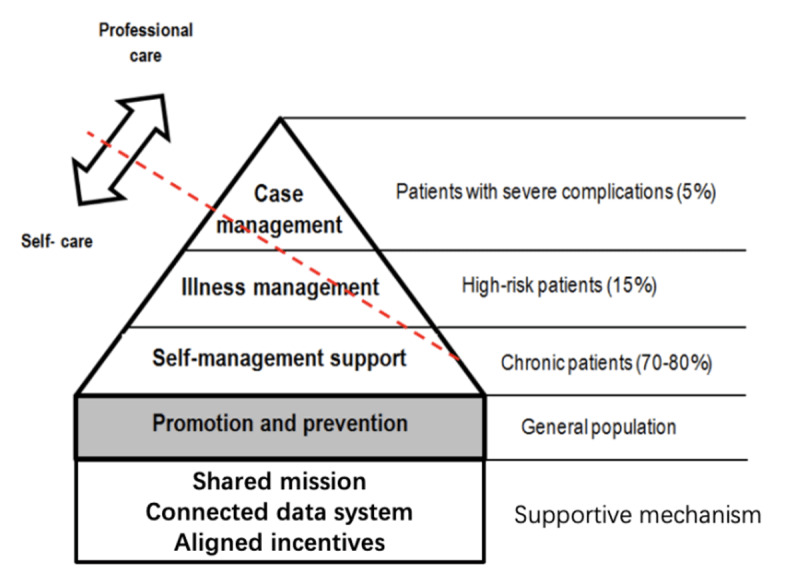
Kaiser Permanente Model.

The Kaiser Permanente (KP) model stands out as a widely recognized integrated care approach, acclaimed for its positive impact on members’ health and the efficient utilization of health resources [20,21]. This model strategically addresses population health by categorizing members into four distinct intervention levels (Figure 3). Firstly, the general population benefits from promotion and prevention services, aimed at controlling exposure to risk factors. Secondly, the majority of chronic patients receive comprehensive support for self-management involving education, training, and clinical consultation. Thirdly, patients with health safety concerns undergo disease-specific care management and transition programs to navigate vulnerable periods safely. Lastly, complex case management targets high-risk patients or those with complex comorbidities, tailoring interventions to meet their specific health goals [1,22].

The realization of coordinated care requires the support of multiple mechanisms, including connected data system, shared mission, and aligned incentives [23]. Data from various sources, such as emergency services, pharmacies, and laboratory tests, are utilized to analyze residents’ health and healthcare needs, thereby achieving population segmentation [24]. Shared mission and consistent incentive mechanisms ensure that healthcare professionals at different levels of care can collaborate with each other, always prioritizing residents’ well-being in the diagnosis and treatment process [4,20].

While the segmentation of the population relies on retrospective data, which may not perfectly align with current needs, the assessment of population health reflects enduring characteristics that tend to remain stable. In contrast, the evaluation of complex case management needs is conducted promptly to address individual variables [24,25]. Additionally, the connected data system, a pivotal support element, is challenging to achieve in alternative contexts, directly impacting managerial and clinical decision support. Therefore, organizations seeking to learn from this model should prioritize the feasibility of the interconnection of their information system.

Based on the KP model, Yuhuan First CIHO has been designing targeted and continuous health services for people with different needs. A wide range of stakeholders including local policymakers, health managers in THCs, medical staff, social workers, and patients were invited to participate in the construction process through seminars, hospital open days, patient activities, etc. Research teams from different universities were involved in 2019 and 2022 respectively to conduct systematic evaluations of CIHO construction. Through data analysis and questionnaire surveys of residents and medical staff, the effectiveness of various measures was objectively demonstrated, and targeted suggestions were provided for further improvement.

## Key Elements of the Strategy

**Figure 4:**
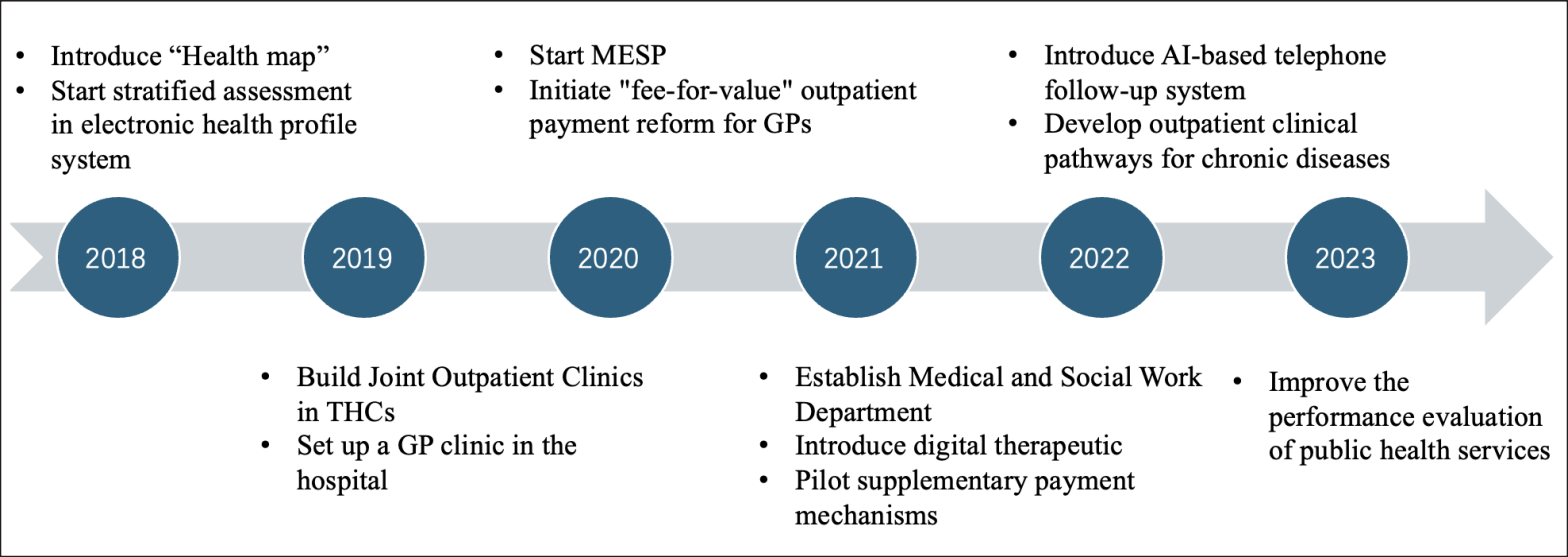
Timeline of main elements of integrated care practice.

Table 1 and Figure 4 show the key strategies and the timeline of the integrated care practice.

**Table 1:** The approaches to integrating care in Yuhuan First CIHO.


DIMENSIONS	KEY STRATEGIES

Promotion and prevention	THCs conduct basic public health services (e.g. chronic disease management, physical examinations, vaccination, and health education). Part-time grassroots personnel have been enlisted to reinforce the manpower and capabilities for health promotion efforts. A Health Map, based on residents’ health statistics, is introduced to analyze the top prevalence diseases in different towns. Activate disease screening and vaccination programs according to the Health Map. Build Joint Outpatient Clinics in THCs to strengthen the screening abilities of General Practitioners (GPs) according to the Health Map.

Self-management support	GPs conduct scheduled follow-ups for chronic disease patients, to provide assistance in managing their living habits, diet and exercise routines. A Medical and Social Work Department was established to conduct supportive care for outpatients and inpatients in both the hospital and THCs. Encourage patients to engage in digital therapeutics, which facilitates self-management through features such as data monitoring, reminders, and alerts.

Disease management	Multidisciplinary teams, comprising professionals such as doctors, nurses, pharmacists, nutritionists, and others, have been established and are dedicated to delivering patient-centric treatments. Platforms (e.g. disease management centers, medical consortium) are utilized to improve specialty capabilities in both the hospital and THCs. Specialist-GP joint clinics have been established in THCs, and a family doctor clinic has been set up within the hospital as transitional measures to guide residents in seeking healthcare in an organized manner. Rehabilitation services are strengthened at the community level.

Case management	Medical Extended Service Package (MESP), which aims at tailored disease management and is provided by medical staff within the CIHO, is launched to provide transitional care after discharge.

Information system reform	The electronic health profile system enables stratified assessment and can formulate standardized follow-up plan. Referral recommendations are prompted automatically in specific circumstances. Automated reminder functions and outpatient clinical pathways were embedded in electronic medical record system. A follow-up system can support medical staff in creating categorized outpatient management plans which can be shared within the management team. Meanwhile, the function of AI-based telephone follow-up was introduced to improve work efficiency.

Payment reform	Initiate “fee-for-value” outpatient medical insurance payment reform for GPs. Combined payment methods were applied for inpatients, including Diagnosis-Related Group (DRG) payment method, payment based on per diem rates, a two-way referral incentive mechanism. Piloted supplementary payment mechanisms by providing chronic disease health insurances. Start performance appraisal for acute cardiovascular and cerebrovascular (death) events.

Shared culture	A unified mission and vision were defined at the establishment of the CIHO. Integrating the concept of health-centered care into the workflow of chronic disease management when transforming the delivery system of healthcare.


### Promotion and prevention

In China, the government procures fundamental public health services, such as chronic disease management, physical examinations, and vaccinations, from THCs. In addition to this, Yuhuan First CIHO has implemented two supplementary strategies to enhance preventive and promotive efforts.

Firstly, the Yuhuan Health Bureau has introduced a ‘Health Map’ initiative to bolster precise health management. This project integrates health-related data from various sources, including healthcare institutions, electronic health record systems, referral platforms, Centers for Disease Control and Prevention (CDC), and the public security system. The Health Map incorporates over 30 data analysis models, encompassing health indices, three-color health grading, and referral evaluations. Through the comprehensive aggregation and analysis of health data, the Health Map can predict regional disease trends, aiding in management decision-making and policy formulation. This includes resource allocation, activation of disease screening, and vaccination programs. By the end of 2022, Yuhuan has initiated ten screening programs for prevalent diseases, providing free vaccinations for pneumonia and influenza, benefiting nearly 60,000 residents. Among them, 2,074 were identified as high-risk individuals, and 93 were diagnosed with cancer. All residents with positive results received subsequent treatment and health management.

Secondly, following the relaxation of the two-child policy, former health and family planning workers have transitioned into community health roles, leveraging their grassroots experiences. After three years of training, they now assume responsibilities for health education and promotion. The inclusion of community health workers not only helps alleviate the shortage of primary health workers to some extent but also crucially fosters a connection between GPs and residents.

### Self-management support

Despite GPs offering regular follow-up visits and healthy lifestyle guidance to contracted residents, the challenge persists for many residents to effectively self-manage diseases due to insufficient health literacy. To address this, Yuhuan First CIHO has forged collaborations with social worker organizations to deliver vital self-management support to residents.

In 2021, Yuhuan First CIHO established the Medical and Social Work Department, tasked with conducting self-management assessments for outpatients at the Metabolic Management Center (MMC). The department conducts monthly, quarterly, and semi-annual follow-ups after enrollment, actively participating in multidisciplinary outpatient consultations to collaborate with medical staff in providing bio-psycho-social comprehensive care. This approach aims to enhance treatment compliance. For inpatients, the department offers emotional support, financial assistance, and organizes humanistic activities. At the community level, they integrate medical resources and provide free healthcare activities to the community. The department also offers home clinic services for socially disadvantaged individuals with limited mobility. Additionally, self-management groups for diabetes patients have been established, organizing group activities to fortify social support.

Meanwhile, Yuhuan First CIHO has entered into partnerships with information technology companies to offer digital health products that empower residents in self-management. For instance, the blood lipid management product facilitates real-time transmission of patients’ heart rates to software through a heart rate monitor or armband. Healthcare professionals can monitor, manage, and process the data through the software, engaging in real-time communication with patients to adjust exercise prescriptions as needed. This digital product significantly contributes to improving patient compliance with exercise interventions, thereby slowing the onset and progression of chronic diseases and achieving better overall outcomes.

### Disease management

Yuhuan First CIHO is strategically enhancing disease management capabilities through three key approaches, steering the focus from disease-centered to people-centered services.

Firstly, a comprehensive integration of disciplinary resources within the hospital is undertaken, continually optimizing the multi-disciplinary team mechanism. Beyond traditional clinical and medical imaging professionals, the inclusion of nurses, pharmacists, and nutritionists is prioritized. This diverse team provides specialized care, medication, and dietary instructions, aiming for improved health outcomes and enhanced inpatient experiences.

Secondly, the organization maximizes industry resources and platforms to improve specialty capabilities. The establishment of a medical consortium and cross-regional specialty alliances facilitates targeted support from advanced tertiary hospitals. This collaboration extends to clinical practices, medical teaching, and research, updating disease treatment and management practices. Medical centers, such as the MMC and Pulmonary and Critical Care Medicine, connect with national academician teams, propelling disciplinary development to a higher echelon. Concurrently, efforts are directed at improving community specialty abilities. Hospital specialists are deployed to THCs to enhance the diagnosis and treatment of prevalent diseases, with a focus on early identification and intervention of critical illnesses. This involves initiatives like specialist-GP joint clinics, joint wards, and training programs.

Thirdly, the organization maintains a steadfast commitment to implementing and promoting two-way referral systems. Initial steps involve creating a comprehensive two-way referral disease catalog, standardizing disease diagnosis and treatment, and offering specialty training programs. Specialist-GP joint clinics in THCs are established based on regional disease spectra identified by the Health Map. Simultaneously, a GP clinic is set up within the hospital to provide general practices. Recognizing the hospital as the primary choice for most residents in medical needs, the GP clinic encourages residents to seek daily treatments at THCs. Besides, rehabilitation services at the community level are continually strengthened. A professional rehabilitation team, including specialists and technicians, is shared within the CIHO to address the shortage of rehabilitation staff in communities. Joint wards and standardized treatment plans are implemented to encourage the transfer of stable inpatients to THCs for ongoing treatment, optimizing the efficiency of health resource utilization.

### Case management

The innovative MESP has been introduced to offer transitional care services following a patient’s discharge. The design of MESP takes into consideration the patient’s disease status, complications, self-management ability, and family support, among other factors, with the aim of ensuring a smooth transition to daily life. MESP is delivered by a multidisciplinary team comprising specialists, nurses, and primary health workers, with additional medical staff included as necessary. Each team member has clearly defined roles and synchronous access to patient health records, facilitating collective oversight of patient health management. Taking the stroke MESP as an illustration, after a patient is discharged, primary health workers arrange home visits as needed to provide guidance on living conditions, diet, and exercise. A specialist nurse conducts monthly follow-up calls to assess the recovery progress of patients and reminds them of their follow-up appointments. Specialized physicians perform pacemaker programming checks and post-operative risk assessments at 1–, 3–, 6–, and 12-month intervals post-surgery. This comprehensive approach ensures that patients receive tailored and continuous support throughout their recovery journey.

### Information system reform

The integration of healthcare services is significantly bolstered by robust support from an information system [26,27], and Yuhuan First CIHO places a key emphasis on seamlessly integrating health information from diverse sources to address the information needs across various stages of health management.

The electronic health record system plays a pivotal role, enabling primary health workers to input residents’ health information. After a stratified assessment for five major chronic diseases, including hypertension, diabetes, stroke, etc., the system formulates standardized follow-up management plans. In instances of unsatisfactory disease management after two consecutive follow-ups, the system automatically recommends patient referral to the hospital for further treatment.

Moreover, outpatient electronic medical record systems are equipped with automated reminder functions. When prescribing medication for chronic patients, the system prompts clinicians with information on clinical risks, individualized treatment goals, and healthy lifestyle recommendations. This encourages the adoption of a ‘dual prescription’ system, addressing both medical and health objectives. Collaborating with the Yuhuan Health Insurance Bureau, an information platform for outpatient clinical pathways for chronic diseases was developed. This platform automatically generates doctor’s order recommendations for enrolled chronic disease patients, issues reminders for order adjustments when needed, and provides warnings regarding the use of medical insurance funds. This facilitates GPs in conducting standardized disease diagnosis and treatment in accordance with national guidelines while making necessary adjustments based on the patient’s use of medical insurance funds.

Post-discharge, a follow-up system supports specialists in creating categorized outpatient management plans for chronic diseases. These plans can be shared with GPs and other health workers, facilitating collaborative online health management and specialized care. The introduction of an AI-based telephone follow-up function for most discharged patients, coupled with manual follow-ups for those with complex conditions, establishes an efficient and flexible human-machine coupled follow-up system. This approach ensures timely monitoring of the disease course, reduces complications associated with chronic diseases, and delays the onset of severe illnesses.

### Payment reform

The integration of care services is complemented by a robust payment support system, serving as an effective means to regulate healthcare service providers and influence residents’ health behavior [28,29]. Yuhuan First CIHO has implemented various innovative approaches to reform healthcare payment, encompassing aspects from disease prevention to health recovery.

Initially, a collaboration with the Yuhuan Health Insurance Bureau in late 2020 led to the transformation of the outpatient medical insurance payment method for chronic disease patients. The shift from the traditional ‘fee-for-service’ to a ‘fee-for-value’ approach involved prepaying outpatient medical insurance costs to contracted GPs on a per capita basis. The bundled payment standard was increased by 200–300 RMB per capita, derived from the previous year’s reimbursement level. At the year-end, any surplus funds were rewarded to GPs after evaluation. At the community level, a standardized management system for acute cardiovascular and cerebrovascular (death) events was established in 2023, followed by a performance appraisal to measure the work input and health value output of public health services. This incentivized primary health workers to actively provide health education and lifestyle interventions, contributing to effective disease management for chronic disease patients. Simultaneously, this approach ensured the safeguarding of medical insurance funds.

The second initiative introduced a tiered classification payment system for inpatients, incorporating the DRG payment method. This system set reasonable payment weights and fee standards for various DRGs based on historical data calculations. It guided medical staff in standardizing diagnosis and treatment, promoting patient recovery and rehabilitation through multidisciplinary collaboration. Additionally, a per diem rates-based payment system was implemented in community health centers, particularly for inpatients in the recovery or rehabilitation phase. This system aimed to reduce hospital-acquired infections and offered greater convenience and affordability for patients. Alongside these inpatient payment reforms, a two-way referral incentive mechanism was introduced based on disease difficulty coefficients, encouraging a tiered approach to inpatient diagnosis and treatment.

Thirdly, Yuhuan First CIHO pioneered supplementary payment mechanisms for chronic disease management by collaborating with commercial insurance companies. Recognizing the role of private health insurance in meeting diverse medical and health needs [30,31], the CIHO collaborated to establish health insurance products. For instance, the ‘Diabetes Care’ program targeted individuals in the pre-diabetic and Type 2 diabetes population. It provided exclusive health records for insured beneficiaries, formulated targeted blood sugar control goals based on their health characteristics, and offered continuous online education and guidance over a year. This approach assisted customers in adopting healthy dietary and exercise habits, guiding them towards achieving health goals. In case of hospitalization or other medical expenses during the coverage period, corresponding reimbursement amounts were provided.

### Shared culture

The effectiveness of all the aforementioned measures hinges on the establishment of a shared organizational culture [32,33]. At the inception of the CIHO, a unified mission was defined: enhancing comprehensive health assurance capabilities for the public. Recognizing that a shift in ideology is a complex and gradual process, we have strategically chosen to initiate the transformation by focusing on prevalent chronic diseases that affect large population groups.

Our approach involves a redesign of the healthcare delivery system, leveraging the restructuring of service workflows to facilitate communication among different disciplines and between specialists and GPs. The incorporation of the concept of health-centered care into these processes, coupled with robust information system support and payment method reforms, aims to boost the motivation of healthcare professionals. We anticipate that as the effectiveness of resident health management gradually unfolds in the future, healthcare professionals will experience a stronger sense of accomplishment. This, in turn, will contribute to the gradual recognition and implementation of health-centered healthcare services, fostering a culture that prioritizes comprehensive health assurance.

## Achieving Results

### Disease burden further alleviated

In 2019, a research team from Fudan University conducted an evaluation of chronic disease management in Yuhuan and the study indicated higher continuity of care was significantly associated with lower healthcare costs [34]. In 2022, this team conducted another comprehensive phase evaluation of Yuhuan CIHOs construction. Utilizing statistical analysis of medical insurance data and hospital electronic medical records spanning from 2016 to 2022, the study revealed significant positive trends (the research findings are under publishing process). Since the establishment of CIHOs, there has been a notable deceleration in the monthly growth rates of medical expenses, urban and rural resident medical insurance expenditures, and individual out-of-pocket expenses, with declines of 54.8%, 92.8%, and 78.54%, respectively. Estimations indicate substantial total savings, amounting to ¥683 million in medical expenses, ¥177 million in urban and rural medical insurance expenditures, and ¥509 million in individual out-of-pocket expenses during the period from August 2018 to October 2021. However, due to the relatively short evaluation period, the change trend in the utilization efficiency of the medical insurance fund remains inconclusive, necessitating ongoing monitoring and analysis in future assessments.

### Residents’ health status further improved

In 2023, remarkable progress was observed in enhancing residents’ health status. The standardized management rates for hypertension and diabetes patients were exemplary at 88.54% and 86.96%, respectively. The primary care attendance rate for patients with hypertension and diabetes reached an impressive 87.93%, surpassing pilot requirements by more than 17 percent and ranking at the forefront of Zhejiang Province. Control rates for blood pressure and blood sugar in the managed population exceeded provincial benchmarks at 72.32% and 54.01%, respectively. The incidence rates of cardiovascular and cerebrovascular diseases in 2022 decreased by 4.07% from 2020, reaching 425.15 per 100,000, and falling below the Zhejiang provincial average (436.19 per 100,000). The Basic Public Health Service Project has consistently ranked first in assessments for 17 consecutive years in Taizhou City of Zhejiang Province, showcasing sustained excellence.

### Hierarchical diagnosis and treatment further deepened

In 2023, significant advancements were made in deepening hierarchical diagnosis and treatment. The primary care attendance rate and county-level care attendance rate reached 79.45% and 90.75% respectively. The number of patients referred downward within the CIHO increased by 10.86%, totaling 21,969, while upward referrals reached 1,033. The catalog of diseases eligible for THCs initial diagnosis expanded to 102 from the initial 63. Primary care visits increased by 24.88% year-over-year to 1.53 million, and discharges rose by 80.72% year-over-year to 6,392.

### Overall hospital technical capabilities further enhanced

In 2023, Yuhuan People’s Hospital demonstrated remarkable enhancements in overall technical capabilities. The number of DRGs, Case Mix Index (CMI), and the number of level three and four surgeries increased by 49.35%, 19.30%, and 21.53% compared to 2018, respectively (data released by the Zhejiang Provincial Health Commission). Notably, in the National Secondary Public Hospital Performance evaluation, Yuhuan People’s Hospital ranked first in Zhejiang province in 2019 and secured the first-place nationwide position in 2021. In 2022, Yuhuan People’s Hospital successfully advanced to a tertiary hospital.

Furthermore, Yuhuan First CIHO has been recognized with multiple provincial and national honors. These include being acknowledged as an outstanding case of medical associations at the provincial level in 2022, achieving the highest grade of ‘A++’ in the initial round of the national assessment for medical associations. The CIHO has also been designated as a national training base for chronic disease management and recognized as one of the first national demonstration units for the collaborative management system of chronic diseases (6 units nationwide). It has further earned the distinction of being one of the first county-level chronic disease management centers (17 units nationwide) and one of the 35 national innovation integration pilot units for the prevention and control of major chronic diseases, standing as the only county-level hospital nationwide. Wenzhou Medical University has established the County-level Chronic Disease Health Management Research Institute in Yuhuan. Moreover, the hospital has become the leading unit for the County-level Chronic Disease Health Management Branch of the China Health Promotion and Education Association.

## Discussion

The Kaiser model offers valuable insights for the efficient delivery of healthcare services to the local population with diverse health needs. However, careful attention must be given to four key aspects during implementation.

Firstly, it is crucial to appropriately, effectively, and dynamically segment the served population. Various segmentation tools, relying on data such as comprehensive electronic medical records, population characteristics, and social data, stratify the population into different segments (ranging from 4 to 269) to address diverse healthcare needs [24]. Consequently, a CIHO needs to thoroughly assess its data integration capabilities and the applicability of segmentation tools. This ensures that available healthcare sources align with population needs, leading to positive health outcomes. The Health Map in Yuhuan integrates data from multiple departments and provides multi-dimensional health data for healthcare managers, medical staff, and residents, thus providing strong data support for population stratification. At present, the stratification models are mainly focused on chronic diseases. In the future, we will continue to develop and apply extra models to support more comprehensive and detailed stratification.

The second challenge involves the allocation of healthcare professional resources. The KP pyramid indicates that most professional care is directed towards patients with severe illnesses and complications, with self-care playing a major role for the general population and most chronic patients. While this principle guides allocation, practical considerations in the context of CIHO must be addressed. Despite the rapid growth in the number of GPs in China in recent years, with 3.28 GPs per 10,000 people in 2022, there is still a relative lack of skills at the grassroots level, leading to insufficient trust from residents [35,36]. This has resulted in the need for specialists’ assistance in daily work [37]. Moreover, health literacy plays a crucial role in developing self-management skills [38,39]. In cases where residents have insufficient health literacy, primary health workers must invest more effort in health promotion and tailor their approach accordingly [40,41]. Yuhuan First CIHO takes a proactive approach by dispatching specialized medical and nursing personnel based on the Health Map to assist primary health workers in disease management. Additionally, the CIHO collaborates with social workers and community health workers to augment health promotion and education efforts. In addressing seasonal needs, a strategic resource allocation plan becomes essential. For instance, intensifying vaccination and public health education before the flu season, along with the establishment of special flu clinics and enhanced critical care services, better meets the residents’ medical attention needs during this period [42,43]. These strategies necessitate a temporary reallocation of human resources. Therefore, in population health management, adjustments to the allocation of resources, represented by the red straight line (Figure 3), should be made while considering factors such as health workforce capabilities, residents’ health literacy levels, seasonal patterns, and other dynamic factors.

The third issue revolves around providing high-quality healthcare services at each level. The fee-for-service mechanism historically led hospital specialists to focus more on treatment, while government-funded primary health workers primarily performed preventive functions [19]. This resulted in the parallel operation of curative and preventive work without effective coordination [44]. Consequently, case management and self-management support were relatively weak compared to illness management and promotion and prevention. Within the context of the Healthy China Initiative, a key challenge in developing CIHOs is effectively integrating existing human resources across different levels to provide high-quality services to residents with different health needs, thereby bridging the gap between curative treatment and preventive care [45]. Yuhuan First CIHO uses various methods to improve the performance of case management and self-management support. In addition to the medical insurance payment reform (e.g. DRG payment) nationwide, we piloted a package payment mechanism of chronic disease patients for contracted GPs, accompanied with a performance appraisal mechanism of health outcomes, to enhance the case management of chronic disease patients. Meanwhile, MESP provides transitional care for specific diseases to improve the continuity of health services. In addition, the introduction of MESP, social workers and private health insurance products not only meets diverse health needs, but also has continuously strengthened residents’ awareness of being the primary person responsible for his/her own health so as to improve motivation for self-management.

The fourth issue concerns the appropriate division of work among specialists, primary health workers, and specialized public health institutions, such as the CDC. The implementation of MESP highlights this challenge. In the early stage of MESP, primary health workers focus on follow-up tasks and are inadequate for disease management. Due to insufficient training in general practice, most specialists primarily concentrate on treatment during GPs’ training, neglecting guidance on providing comprehensive health management [46]. This results in a gap between clinical training and community work for primary health workers [47,48]. To solve this problem, we continuously strengthened the health management training for specialists and disease-specific training for GPs, thus improving team cooperation and service continuity. Meanwhile, we integrate social work organizations, and digital therapeutics to enhance the effectiveness of curative and preventive integration. As of 2023, social workers have formulated “Guidelines for Medical and Social Work Services for Diabetes Patients”, currently undergoing institutional approval. A project involving social workers’ participation in diabetes management under the context of CIHO is also in progress, awaiting subsequent results for further application. Public health workers currently have limited integration in this process, and effective cooperation methods need further exploration.

## Conclusion

Guided by the population health management principles of the Kaiser Pyramid, Yuhuan First CIHO has embarked on a multifaceted approach encompassing health prevention and promotion, self-management support, disease management, and case management. In tandem, corresponding reforms have been instituted in information systems, payment methods, and cultural integration. This holistic strategy has produced positive outcomes, bolstering technical capabilities, advancing the hierarchical diagnosis and treatment system, improving residents’ health status, and mitigating the economic burden of diseases. Areas necessitating ongoing optimization and enhancement in future practices include rational population segmentation, effective allocation and collaboration of healthcare human resources, and the refinement of the health management process across the entire population.
